# Association Between Body Mass Index and Response to Disease-Modifying Therapies in Patients With Relapsing-Remitting Multiple Sclerosis at King Abdulaziz University Hospital: A Retrospective Study

**DOI:** 10.7759/cureus.32695

**Published:** 2022-12-19

**Authors:** Mohammed N Aljehani, Ziyad I Alshehri, Faisal A Alharbi, Yaser T Balbaid, Abdullah M Wali, Alaa A Alotaibi

**Affiliations:** 1 Medicine, King Abdulaziz University, Jeddah, SAU; 2 Neurology, King Abdulaziz University Hospital, Jeddah, SAU

**Keywords:** disease modifying drugs, obesity, demyelinating disorders, relapsing-remitting multiple sclerosis, all neurology

## Abstract

Background

Multiple sclerosis (MS) is an immune-mediated inflammatory disease that attacks myelinated axons in the central nervous system, destroying the myelin and axon to varying degrees and producing significant physical disability. So far, many studies have found that having a high body mass index (BMI) is associated with severe autoimmune and neurodegenerative disease course. However, the impact of BMI on disease-modifying therapy (DMT) response in terms of decreasing relapses and improving overall health remains unknown.

Aims and objectives

The study aimed to demonstrate the effect of BMI on DMT responsiveness in patients with relapse-remitting MS at a tertiary hospital.

Methods and material

A single-center retrospective study was conducted at a tertiary care center in Jeddah, Saudi Arabia. The study included 89 individuals with relapsing-remitting MS who had their BMI measured within six months of their first clinical relapse, as well as their clinical response to the DMT (number of relapses on a single DMT after six months of initiation) and MRI changes (development of new T2 lesions or gadolinium-enhancing lesions on single DMT six months after DMT initiation).

Results

Demographic data revealed a female predominance of 71.9%, and 51.7% of the patients had a normal weight. The most commonly prescribed DMT was Gilenya at 47.2%. A significant relationship was found between BMI and the total number of clinical relapses (p=0.038), with the co-existence of a positive correlation between BMI and the number of relapses after at least six months of initiation of DMT. Additionally, MS patients who had both positive MRI changes and obesity had a significantly higher BMI mean than non-obese.

Conclusion

Increased BMI appeared to be associated with a lower response to DMT, as overweight patients had a worse course than normal and underweight patients. Pharmacokinetic differences are the most likely factors implicated in medication responsiveness.

## Introduction

Obesity and overweight are on the rise throughout the world, with the most noteworthy increases in children and adults over the last three decades [1]. Maintaining a healthy weight is critical for preventing various health problems (such as type 2 diabetes mellitus, dyslipidemia, and coronary artery disease) and promoting good aging. It can also help with symptom control and disability prevention in chronically unwell people with diseases such as multiple sclerosis (MS) [2-4].

Body mass index (BMI) is a statistical metric used to calculate body fat in people of all ages. BMI is calculated by multiplying a person's weight in kilograms by their height in meters squared through the following equation: weight (kg)/ height^2^ (m^2^) [5].

MS is becoming more common and prevalent worldwide, especially in traditionally low-prevalence areas, with women having a greater prevalence than men [6, 7]. MS is an immune-mediated inflammatory disease that damages myelinated axons in the central nervous system, causing variable degrees of myelin and axon damage and significant physical disability in more than 30% of people within 20-25 years [8].

Disease-modifying therapies (DMTs) are a group of medications that can help with the symptoms and progression of MS [9]. DMTs are the gold standard of treatment for people with relapsing-remitting MS (RRMS) who are still early in the disease cycle [10,11]. DMTs can modulate inflammatory responses by affecting lymphocyte counts, subsets, activation, and localization [12]. Patients with RRMS, as well as progressive forms of MS relapse, may benefit from them. A relapse is defined as the onset of new symptoms or worsening of existing symptoms that last at least 24 hours and are separated by at least one month from a previous relapse [9].

Several studies have found a link between metabolic stress, autoimmunity, and neurodegeneration, and increasing BMI in a variety of chronic inflammatory disorders [13]. In RRMS patients, obesity and an altered lipid profile are connected to increased cerebral inflammation and clinical impairment. Increased adipocytokines and lipids can help counteract the negative effects of obesity on RRMS progression [14]. Individuals with a BMI classified as overweight or obese have a lower chance of achieving complete remission and no evidence of disease activity status, according to a prior prospective analysis of 86 patients with RRMS [15].

Despite the foregoing, no research in Saudi Arabia has investigated the impact of BMI on DMT responsiveness in MS patients. Therefore, this study aimed to show the influence of BMI on the response to DMTs among patients with RRMS at a tertiary care center in Jeddah, Saudi Arabia.

## Materials and methods

Study design and setting

A retrospective study that reviewed the medical records of 177 adult MS patients between 2010 and 2021 was conducted from May 2021 to July 2021 at King Abdulaziz University Hospital (KAUH), Jeddah, Saudi Arabia.

Study participants

Patients of both sexes aged 18 and above were chosen only if they met the following criteria: final diagnosis of MS as defined in McDonald’s 2017 criteria [16], a period of six months or more from the initiation of DMTs, a well-documented height and weight measurement within six months of first clinical relapses, number of clinical relapses throughout the illness duration, and magnetic reasoning image (MRI) results. Patients who were documented as uncommitted to the medications and those who were included in an experimental study were excluded. Patients with a period of less than six months from the initiation of DMTs were excluded from the study, leaving 89 patients as study participants.

Data collection

The information used from the medical records was age, gender, height, weight, BMI, time of first clinical relapses, duration of illness, the total number of clinical relapses, type of DMT used, treatment duration of DMT, clinical response to the DMT (number of relapses on a single DMT after six months of initiation), and MRI changes (development of new T2 lesions or gadolinium-enhancing lesions on single DMT six months after DMT initiation).

Body mass index

The BMI was categorized by the World Health Organization as follows: BMI below 18.5 indicates underweight, 18.5-24.9 indicates normal weight, 25.0-29.9 indicates overweight, 30.0-34.9 indicates obesity class I, 35.0-39.9 indicates obesity class II, and above 40 indicates obesity class III [17]. 

Disease-modifying therapy

In general, patients were given doses based on the recommendations of the medicine manufacturer.

Interferon beta-Ia is given as 30 mcg injected intramuscularly once a week or 22/ 44 mcg three times weekly subcutaneously. Fingolimod exists as a hard capsule of 0.25 or 0.5 mg, which is given orally once a day. Ocrevus is dosed twice yearly, after two initial doses (initial two doses are 300 mg IV once; repeat dose two weeks later, and the subsequent doses are 600 mg IV every six months). Finally, Rituximab is given as a single IV infusion of 500 or 1000 mg every 6-12 months.

Ethical approval

Ethical approval for the study was obtained from the Research Ethics Committee at KAUH.

Statistical analysis

Data were analyzed using SPSS version 26 (IBM Inc., Armonk, New York), and quantitative data were expressed as mean and standard deviation (mean ± SD). Both Mann-Whitney and Kruskal-Wallis tests were used for non-parametric variables. Correlation analysis using Spearman's test was applied, and a p-value of less than 0.05 was considered statistically significant.

## Results

A total of 177 patient files were reviewed during the study period, of which 89 patients met the inclusion criteria.

Table 1 shows that there were 64 (71.9%) females and 25 (28.1%) males, with a mean age of 33.85 ± 9.26 years and a mean age at diagnosis of 25.15 ± 8.77. The mean BMI of the studied patients was 25.76 ± 6.03 kg/m^2^ and most of them (51.7%) had a normal weight. Forty-two (47.2%) participants used Gilenya (fingolimod) as their current DMT, and 66 (74.2%) had no previous DMT. Almost 21 (23.5%) had MRI changes after using the DMTs, and 4.5% of them had positive MRI findings and obesity at the same time.

**Table 1:** Distribution of studied patients according to their characteristics, current and previous DMT, MRI changes, and coexistence of positive MRI findings and obesity BMI: body mass index, DMT: disease-modifying therapy

Variable	No. (%)
Age	33.85 ± 9.26
Age at diagnosis	25.15 ± 8.77
BMI categories	
Underweight	3 (3.4)
Normal weight	46 (51.7)
Overweight	21 (23.6)
Obese	19 (21.3)
BMI (mean ± SD)	25.76 ± 6.03
Gender	
Female	64 (71.9)
Male	25 (28.1)
Current DMT used	
Betaferon (interferon beta-1b)	23 (25.8)
Gilenya (fingolimod)	42 (47.2)
Ocrevus (ocrelizumab)	3 (3.4)
Rituximab	21 (23.6)
Previous DMT used	
Betaferon (interferon beta-1b)	19 (21.3)
Gilenya (fingolimod)	2 (2.2)
None	66 (74.2)
Rituximab	2 (2.2)
MRI changes (development of new T2 lesions or gadolinium-enhancing lesions on single DMT after six months of DMT initiation)	
No	68 (76.4)
Yes	21 (23.6)
MRI and obesity	
Positive MRI finding and obese	4 (4.5)
Negative MRI and non-obese	85 (95.5)

Table 2 illustrates that the mean duration of illness in years was 6.69 ± 5.6. In addition, the mean duration of the current DMT used, the total number of clinical relapses, and the number of clinical relapses after six months of using the DMT were 3.07 ± 3.18 years, 3.35 ± 2.5 relapses, and 0.77 ± 1.2 relapses, respectively.

**Table 2:** Statistical analysis of clinical information DMT: disease-modifying therapy, GD: gadolinium, OCB: oligoclonal bands

Variable	Mean ± SD
Time since the first clinical relapse (time of admitting diagnosis - initial assessment; first neurological symptoms) in years	7.35 ± 6.11
Time of confirmed diagnosis (time of final diagnosis from the system or medical reports, time of 2nd clinical relapses, new MRI lesions or GD enhancing lesions or positive OCB) in years	6.74 ± 6.12
Duration of illness (multiple sclerosis) in years	6.69 ± 5.6
Duration of the current disease-modifying therapy (DMT) used; in years	3.07 ± 3.18
Total number of clinical relapses	3.35 ± 2.5
Clinical response to the DMT (number of relapses on a single DMT six months after initiation)	0.77 ± 1.2

From a BMI perspective, our study showed a significant relationship between BMI and the total number of clinical relapses (Table 3). There was a positive correlation between BMI and the clinical response to DMT (Table 4). Moreover, MS patients who had a coexistence of positive MRI findings and obesity had a significantly higher mean BMI. On the other hand, a non-significant relationship was found between mean BMI and MRI changes (p>0.05) (Table 5).

**Table 3:** Spearman's correlation analysis between variables and the total number of clinical relapses BMI: body mass index, DMT: disease-modifying therapy, GD: gadolinium, OCB: oligoclonal bands

Variable	Total number of clinical relapses	
	r	p-value
Age	0.06	0.572
Age at diagnosis	0.007	0.947
BMI	- 0.2	0.038
Time since the first clinical relapse (time of admitting diagnosis - initial assessment; first neurological symptoms) in years	0.22	0.036
Time of confirmed diagnosis (time of final diagnosis from the system or medical reports, time of second clinical relapses, new MRI lesions or GD enhancing lesions or positive OCB)	0.23	0.034
Duration of illness (multiple sclerosis) since confirmed diagnosis; in years	0.26	0.013
Duration of the current disease-modifying therapy (DMT) used; in years	0.01	0.896

**Table 4:** Spearman's correlation analysis between variables and clinical response to the DMT BMI: body mass index, DMT: disease-modifying therapy, GD: gadolinium, OCB: oligoclonal bands

Variable	Clinical response to the DMT (number of relapses on a single DMT after six months of initiation)	
	r	p-value
Age	0.13	0.233
Age at diagnosis	- 0.18	0.102
BMI	0.03	0.754
Time since the first clinical relapse (time of admitting diagnosis – initial assessment; first neurological symptoms) in years	0.05	0.603
Time of confirmed diagnosis (time of final diagnosis from the system or medical reports, time of second clinical relapses, new MRI lesions or GD enhancing lesions or positive OCB) year entry is needed	0.08	0.462
Duration of illness (multiple sclerosis) since confirmed diagnosis; in years	0.09	0.385
Duration of the current disease-modifying therapy (DMT) used; in years	0.16	0.132

**Table 5:** Relationship between mean BMI and MRI findings and coexistence of positive MRI findings and obesity BMI: body mass index, DMT: disease-modifying therapy

Variable	BMI (mean ± SD)	Mann Whitney test	p-value
MRI changes (development of new T2 lesions or gadolinium-enhancing lesions on single DMT after six months of DMT initiation)		0.6	0.543
No	25.95 ± 6.15		
Yes	25.12 ± 5.72		
MRI and obesity		2.71	0.003
Positive MRI finding and obese	34.42 ± 5.17		
Negative MRI and non-obese	25.35 ± 5.78		

Table 6 shows that a non-significant relationship was found between patients' gender and MRI findings, the coexistence of positive MRI findings and obesity, the total number of clinical relapses, and clinical response to DMT (p<0.05).

**Table 6:** Relationship between variables and patients' gender DMT: disease-modifying therapy

Variable	Female No. (%)	Male No. (%)	Test	p-value
MRI changes (development of new T2 lesions or gadolinium-enhancing lesions on single DMT after six months of DMT initiation)			0.24	0.618
No	48 (70.6)	20 (29.4)		
Yes	16 (76.2)	5 (23.8)		
MRI and obesity			0.02	0.888
Positive MRI finding and obese	3 (75)	1 (25)		
Negative MRI and non-obese	61 (71.8)	24 (28.2)		
Total number of clinical relapses	0.85± 1.35	0.57 ± 0.66	0.15	0.874
Clinical response to DMT	0.85± 1.35	0.57 ± 0.66	0.96	0.064

Table 7 shows that patients on Betaferon (interferon beta-1b) as their current DMT had a significantly higher mean the total number of clinical relapses, while patients on rituximab had a higher mean of clinical relapses after six months of initiation of the DMT but was effective enough to be second to Gilenya (fingolimod) from an MRI perspective and made less new change on MRI. On the other hand, a non-significant relationship was found between the current DMT used and MRI findings or the coexistence of positive MRI findings and obesity (p>0.05).

**Table 7:** Relationship between variables and current DMT used DMT: disease-modifying therapy

Variable	Current DMT drugs				Test	p-value
	Betaferon (interferon beta-1b)	Gilenya (fingolimod)	Ocrevus (ocrelizumab)	Rituximab		
MRI changes					6.31	0.097
No	14 (20.6)	32 (47.1)	3 (4.4)	19 (27.9)		
Yes	9 (42.9)	10 (47.6)	0 (0.0)	2 (9.5)		
MRI and obesity					2.08	0.556
Positive MRI finding and obese	2 (50)	2 (50)	0 (0.0)	0 (0.0)		
Negative MRI and non-obese	21 (25.7)	40 (47.1)	3 (3.5)	21 (25.7)		
Total number of clinical relapses	1 ± 1.68	0.71 ± 1.12	0.001 ± 0.001	0.79 ± 0.78	3	0.018
Clinical response to the DMT (number of relapses on a single DMT after 6 months of initiation)	2.95 ± 1.66	3.21 ± 2.97	1.67 ± 0.57	4.24 ± 2.07	3	0.339

## Discussion

This study aimed to show the influence of BMI on the response to DMTs among patients with MS at King Abdulaziz University Hospital in Jeddah, Saudi Arabia.

According to this study, there was a significant association between BMI and total clinical relapses, which raises the suspicion that BMI plays a role in the process of inflammation and disease progression, thus worsening the prognosis of the patients. Many studies have indicated that a high BMI during adolescence has a positive effect as a precipitating factor for MS onset and progression [18, 19]. Therefore, we recommend that patients lower their BMI, as it has been linked with worse prognoses and other comorbidities.

According to this study, there was a significant association between BMI and poor response to DMT medications while considering the different medications patients used and the variations in their BMIs. This goes hand in hand with other studies of similar objectives; there was a study conducted in Italy that showed that BMI influences the clinical response to ocrelizumab DMT [20], attributing the influence to different pharmacokinetics in high-BMI patients. There was also a study in Germany that found patients with a high BMI had worse responses to first-line treatment than others, although the study was conducted on pediatric patients [21]. No significant association was found between patients' gender and disease activity, measured using the number of relapses and MRI findings. This is consistent with several studies that showed that both genders progress and respond to the disease in a similar manner [22-24]. Although females are more prone to developing MS in the first place, there are no signs showing that they are more or less likely to have a more aggressive form of the disease, which tends to relapse more often. Another study also clearly showed that gender had no significant impact on the age of onset or disease activity as a percentage of the whole sample [25].

Our sample also showed that patients on rituximab had a significantly higher mean of clinical relapses after six months of initiation of DMTs compared to the rest. We do not suspect this to be explained by the drug's ineffectiveness; rituximab is proven to be extremely effective in controlling MS [26, 27]. Although it still requires more trials, its results are promising globally. We attribute this finding to the fact that most patients treated with rituximab had advanced stages of the disease, and their cases were quite aggressive compared to patients who received different treatments.

There were multiple limitations to this study, most of which were poor documentation and a lack of knowledge and awareness among patients, which made it difficult to find multiple BMI records and MRI results. We also had to lower the number of patients studied due to insufficient data.

## Conclusions

According to this study, increased BMI appears to be associated with a weaker response to DMTs, as overweight patients had a worse response than normal and underweight patients. Differences in pharmacokinetics are probably the factor implicated in response to the drugs. We suggest that achieving a normal healthy weight be added to the treatment regimen to optimize the drug's efficacy or that the dosage is adjusted for patients of different BMIs to achieve better results in treatment.
