# Correction: Association Between Body Mass Index and Response to Disease-Modifying Therapies in Patients With Relapsing-Remitting Multiple Sclerosis at King Abdulaziz University Hospital: A Retrospective Study

**DOI:** 10.7759/cureus.c88

**Published:** 2023-01-03

**Authors:** Mohammed N Aljehani, Ziyad I Alshehri, Faisal A Alharbi, Yaser T Balbaid, Abdullah M Wali, Alaa A Alotaibi

**Affiliations:** 1 Medicine, King Abdulaziz University, Jeddah, SAU; 2 Neurology, King Abdulaziz University Hospital, Jeddah, SAU

This article has been corrected to fix a typo in the first sentence of the Results section:

"A total of 177 patient files were reviewed during the study period, of which 87 patients met the inclusion criteria."

The correct amount of patients meeting the inclusion criteria was in fact 89, not 87. This sentence has been amended to reflect this. The journal sincerely regrets that this typo was not caught and corrected prior to publication.

